# Nonverbal Social Sensing: What Social Sensing Can and Cannot Do for the Study of Nonverbal Behavior From Video

**DOI:** 10.3389/fpsyg.2021.606548

**Published:** 2021-07-27

**Authors:** Laetitia Aurelie Renier, Marianne Schmid Mast, Nele Dael, Emmanuelle Patricia Kleinlogel

**Affiliations:** Department of Organizational Behavior – Faculty of Business and Economics (HEC), University of Lausanne, Lausanne, Switzerland

**Keywords:** nonverbal behavior, social sensing, coding, extraction, communication, technology, annotations, algorithm

## Abstract

The study of nonverbal behavior (NVB), and in particular kinesics (i.e., face and body motions), is typically seen as cost-intensive. However, the development of new technologies (e.g., ubiquitous sensing, computer vision, and algorithms) and approaches to study social behavior [i.e., social signal processing (SSP)] makes it possible to train algorithms to automatically code NVB, from action/motion units to inferences. Nonverbal social sensing refers to the use of these technologies and approaches for the study of kinesics based on video recordings. Nonverbal social sensing appears as an inspiring and encouraging approach to study NVB at reduced costs, making it a more attractive research field. However, does this promise hold? After presenting what nonverbal social sensing is and can do, we discussed the key challenges that researchers face when using nonverbal social sensing on video data. Although nonverbal social sensing is a promising tool, researchers need to be aware of the fact that algorithms might be as biased as humans when extracting NVB or that the automated NVB coding might remain context-dependent. We provided study examples to discuss these challenges and point to potential solutions.

## Introduction

Investigating nonverbal behavior (NVB), and in particular kinesics, namely face and body motions used in communication (Birdwhistell, 1955; Burgoon and Dunbar, 2018), involves observing social interactions and coding movements of participants in the face and the body. Manually coding NVB takes a considerable amount of time and resources because it means having coders sit in front of a video screen and, for instance, count the frequency of smiles, calculate the duration of gazing, code interruptions, or rate the target on a more global judgment (e.g., how dominant or deceiving) for many hours over many days. Moreover, this does not include the additional work of training the coders and establishing reliability among them.

Due to advanced growth in computer vision, new technologies and approaches (e.g., SSP, Vinciarelli et al., 2009a,b, 2012) have been developed to use and train algorithms to code NVB as action/motion units or as more global judgments (inferences) from videotaped individuals in social interactions (e.g., trustfulness). This has given rise to nonverbal social sensing, an approach that allows to automatize most of the NVB coding.

Once such algorithms are developed, they have the advantage of being scalable. Therefore, to the extent that researchers code the same NVB or judge the same inferences in different studies, such algorithms are valuable to researchers. Moreover, there is no standardized codebook detailing exactly how to code NVB (e.g., should smiling be assessed as a frequency, a duration, or a general impression about how much a person smiles on a scale of 1–5), which makes the comparison of results pertaining to NVB difficult across different studies. If more researchers used nonverbal social sensing, this field might gain in standardization and we might discover new insights that were not previously possible since the different coding methods would introduce too much noise to detect the signal. Furthermore, using nonverbal social sensing, when studying NVB, has the potential to reveal meaningful nonverbal patterns more easily (e.g., looking at the interaction partner while speaking, see Burgoon et al., 2014 for an example in detection of deception using computer-assisted coding and an algorithm to identify temporal patterns) instead of extracting only isolated NVB cues (e.g., duration of looking at the interaction partner and the number of speech turns of the target). These advantages might attract new researchers to study NVB, thus enriching and broadening the field.

The aim of this paper is to provide information and guidance to researchers who consider using nonverbal social sensing for their studies. We explained how nonverbal social sensing works, where we see the challenges of using it for the study, and how we recommend addressing such challenges. We illustrated these aspects with selective study examples.

In this paper, we focused on kinesics and the use of nonverbal social sensing based on video recordings (see Poppe, 2017 for an application of nonverbal social sensing beyond video recordings). Kinesics refers to two categories of NVB: (1) gesture and posture and (2) face and eye behavior (Vinciarelli et al., 2009a; the latter is also referred to as gaze, Harrigan, 2005, p. 137). Moreover, we focused on the extraction of NVB or inferences based on videotaped targets. We did not consider the sensor-based technologies, which require participants to wear sensors that register their NVB during the interaction task (see, e.g., Poppe et al., 2014; Rahman et al., 2019).

## The Level of Nonverbal Coding: Unit vs. Inference

We studied the NVB coding on two different levels: action/motion units and kineme/inferences. An action/motion unit refers to specific body motions, such as muscle movements in the face and frequency or duration of a specific NVB (e.g., head motion and movement of the lips) or in the body (e.g., arm movement and leaning). As for “micro-kinesics,” these units do not carry social meaning (see Birdwhistell, 1952). However, researchers are interested not only in specific nonverbal cues but also in inferences and the coding of global judgments based on NVB. Coders make inferences about trustworthiness, hireability, charisma, personality, or motivation of a target by observing the behaviors of the participant.

The lower the level of abstraction in coding, the more the interpretation of what the behavior means is already included in the coding, whereas higher levels of abstraction need interpretation and information about the context (see Birdwhistell, 1970). To illustrate, the number of smiles does not have much meaning attached to it. The meaning of smiling depends largely on the context. For instance, the simulation-of-smiles model (Niedenthal et al., 2010; Rychlowska et al., 2017) proposes to distinguish smiles according to their roles as follows: the smile that communicates positive emotions (enjoyment smile), the smile that suggests positive social intentions (affiliative smile), and the smile that reflects status or control (dominance smile). However, coding friendliness for instance (which might be based on smiling, but not exclusively) involves coding the meaning of the underlying NVB (e.g., smile, eye contact, and voice tone) to decide to what extent an individual appears friendly.

In summary, action/motion units can be coded relatively objectively, whereas inferences are more subjective because they need interpretation and are more context-dependent. This distinction between units and inferences, between objective and subjective measurements (Burgoon and Dunbar, 2018), is key in understanding the workings and challenges of nonverbal social sensing.

## How Nonverbal Social Sensing Works

Nonverbal social sensing originates in the field of SSP. SSP aims at automatically analyzing and synthesizing social signals (Vinciarelli et al., 2009b). SSP allows transforming raw input data (e.g., video recordings of people in social interactions) into social signals (i.e., units or inferences). Developing algorithms for nonverbal social sensing requires input data (i.e., videos of participants and ground truth). The videos refer to the material on which the algorithm is trained to extract and classify the NVB. The ground truth refers to the labels (e.g., manual coding or self-report) used as the standard of extraction or classification. The ground truth is either collected for the entire dataset or only on a subset (i.e., training set) of videos.

Ground truth data can be obtained in many different ways. For instance, satisfaction ratings of clients of a call center have been used as ground truth to train an algorithm to predict client satisfaction based on vocal cues of the call center employees (Zweig et al., 2006; Segura et al., 2016). When wanting to develop an algorithm that extracts personality, self-reports or other reports of personality can be used as the ground truth or expert assessments. When interested in developing algorithms that mimic human perception and judgment (e.g., perceived trustworthiness and hireability), we required human coders who are instructed and trained to perform the coding manually (i.e., manual annotations serve as the ground truth) or naïve raters who report their perception of the targets (e.g., source credibility ratings, Pentland, 2018).

We present below the general functioning of nonverbal social sensing in the following sections. We first present the application to NVB studies at the unit level. Second, we present two approaches to address NVB at the inference level.

### Nonverbal Social Sensing at the Unit Level

At the action/motion unit level, nonverbal social sensing allows capturing a wide variety of nonverbal cues, such as micro-expressions, gestures, and movements. To illustrate, in the case of micro-expressions, the coding consists of extracting the frequency and the duration of muscle movements in the face, such as in the study of facial expressions. One of the most well-known and used classification methods to manually code facial expressions is the facial action coding system (FACS; Ekman and Friesen, 1978). When using the FACS, human coders note whether a facial action (i.e., activation of facial muscles such as lip corners going up or brow-raising) is present when coding a video. From this coding system, researchers develop algorithms to automatically recognize facial action units (AUs) from still records (Pantic and Rothkrantz, 2004) and moving records (Kapoor et al., 2003; Bartlett et al., 2006; Tong et al., 2006). As an application example, researchers used nonverbal social sensing to study the existence of cross-cultural differences in smiling (AU12) and brow furrowing (AU4) (McDuff et al., 2017). These researchers used automated extraction of these two units to study the effect of culture (i.e., individualist vs. collectivist), setting (i.e., home vs. lab), and gender on facial expressions. Their use of nonverbal social sensing enabled them to observe cultural (e.g., higher rate of brow furrowing in individualist culture than in collectivist culture) and gender differences (e.g., more smiling and less brow furrowing for women than men in both cultures, but more pronounced differences in individualist culture) at a lesser cost and on a larger scale (e.g., using a sample of 740,984 participants across 12 countries). Some of these researchers particularly worked on the development of algorithms for the detection of AU12 and AU4 and on a corpus of data for the study of spontaneous facial expressions (McDuff et al., 2013).

We might also need human coders at the unit level. In order to train an algorithm to extract the number of times a person nods in a video, we need to define which head movements qualify as a nod. This information is typically provided by human coders. We need several independent human coders to watch the same videos and to judge whether a given head movement is a nod, and then, we need to test for reliability (i.e., the extent to which the independent coders are consistent). The machine is then fed with this information together with the corresponding video, and from these two inputs, the machine can learn to detect head nods (e.g., Nguyen et al., 2012). Once trained, the algorithm will have learned to extract the features and classify them as action/motion units and can be used on new datasets. However, instead of measuring the ground truth, researchers might also rely on open-source tools such as OpenPose (i.e., body behavior; Cao et al., 2019) or OpenFace (i.e., face behavior; Baltrusaitis et al., 2018). OpenPose is an open-source library for multi-person detection providing real-time pose estimation (e.g., head, hand, foot, and face). OpenFace is also an open-source library designed to detect facial landmarks (e.g., facial AU, head pose, and eye-gaze). Both libraries are well-recognized tools for coding NVB as action/motion units enabling researchers to skip the training stage of nonverbal social sensing (for an application of OpenFace, see Burgoon et al., 2021).

### Nonverbal Social Sensing at the Inference Level

At the inference level, NVB is coded according to its meaning, starting from the kineme to a higher-order inference. As examples of kinemes, we cite visual dominance—the ratio of the percentage of looking while speaking divided by the percentage of looking while listening (Dovidio et al., 1988)—or visual back-channeling—head nods while listening (Nguyen et al., 2012).

Nonverbal social sensing allows extracting data related to higher-order inferences or global judgments. For example, algorithms can capture how dominant or how trustworthy individuals are perceived through the measure of a combination of NVB (Burgoon and Buller, 1994; Hall et al., 2005; Mast et al., 2011). For instance, researchers used nonverbal social sensing to automatically predict the level of dominance of individuals during group interactions (Jayagopi et al., 2009) or their hireability (Naim et al., 2015). Other instances include the detection of personality traits (e.g., Pianesi et al., 2008; Batrinca et al., 2011), using personality recognition to improve automated detection of deception (An et al., 2018), or the detection of emotions based on body movements (Glowinski et al., 2008).

For higher-order inferences, the following two main approaches are currently pursued. In the first approach, the NVB is extracted automatically from the video input (as described for the motion unit extraction), and this extracted NVB is then linked with the ground truth. The machine is trained to first extract the nonverbal features (e.g., a nod and a smile) and only then learns to link those to the higher-order inferences (e.g., the classification of a target as friendly). For instance, to predict who gets hired for a job, the machine can first extract a set of specific NVB and then link it to the ground truth of hiring decisions. Another example is training a machine to predict social skills or personality (Biel et al., 2013; Muralidhar et al., 2018; Rasipuram and Jayagopi, 2018) or emotions (Ahn et al., 2010) based on previously extracted nonverbal cues. Again, the ground truth has to be measured (e.g., human coders assessing the personality of the people in the video or a self-report of their personality). The machine that extracted the NVB will link the extracted NVB to the ground truth. This approach allows identifying the NVB that is conductive of being hired (Frauendorfer et al., 2014; Nguyen et al., 2014; Muralidhar et al., 2016), which is important for training and the transparency of the decision-making. When predicting that a person is conscientious, this approach allows knowing which NVB pattern is responsible for this prediction.

In the second approach, the machine is fed with the video input and the ground truth (e.g., hireability) and learns to classify the videos into (not) hireable without involving the explicit extraction of NVB. This second approach relies on deep learning (see Mehta et al., 2019 for a review of the use of deep learning in the detection of personality traits). The machine is given the videos and the ground truth, which this time is an inference such as, for instance, how dominant a person behaves in a social interaction rated by external observers or the personality assessed *via* self-report. The machine learns the link between the training videos and the ground truth (i.e., annotated dataset). However, the researcher or user will not know which array of nonverbal cues the algorithm uses for the prediction. Does the machine judge people as dominant because they speak a lot, because of a loud tone of voice, because they move more, or because of their gender or skin color or any combination thereof? There is no way to be certain.

Using nonverbal social sensing for higher-order inferences by either first extracting the NVB or directly linking the videos to the ground truth (i.e., annotated dataset at the inference level) is a choice a researcher needs to make based on how important it is to know which behaviors are responsible for the inference. This approach might be considered less costly because researchers only need to feed the data to the machines without relying on human coders. However, the size of the dataset to be fed into the machine is large (i.e., hundreds of videos) and thus also potentially costly. Thus, the benefits and shortfalls of deep learning depend on the goals of the researchers. If they are interested in determining the behaviors responsible for the inferences, we cautioned researchers when using deep and unsupervised learning approaches given their black-box nature. However, if researchers are primarily interested in higher-order inferences, deep learning appears to be a suitable approach (e.g., Mehta et al., 2019). In between, supervised deep learning might also reduce the black-box aspect associated with unsupervised learning and might lead researchers to discover new patterns of behaviors and inferences (see LeCun et al., 2015). Finally, concerning lower-order inferences, advances in deep learning enable researchers to automatically extract human pose at a lesser cost (Mathis et al., 2018; Arac et al., 2019).

There are some corpora of annotated data concerning higher-order inferences available. For example, corpora of annotated data are available in the domain of group interaction studies (see Gatica-Perez, 2015 for a list of corpus), leadership emergence (corpus cited in Sanchez-Cortes et al., 2011, 2013), psychological distress (Gratch et al., 2014), or personality detection (Mana et al., 2007). These corpora might help reduce the cost of collecting the input data.

## Challenges When Using Nonverbal Social Sensing

Under this section, we highlight key challenges associated with the use of nonverbal social sensing for researchers. We additionally make suggestions to address them.

### The Risk of Bias

Algorithms are often used because people think they are less biased. It is true that once the algorithm runs, it does not make a difference between, for example, women or men showing a certain behavior. It simply codes the behavior, whereas human coders might be affected by the gender of the person showing the behavior they are about to code. However, algorithms are only as good as the ground truth on which they are trained. In other words, if the ground truth is biased, the algorithm will be biased. The risk for biased ground truth is higher for predictions at the inference level than at the unit level because the former is a more subjective coding than the latter. Therefore, collecting ground truth on nodding is probably less biased than collecting ground truth on, for example, the hireability of a person for a job.

Bias might also plague algorithms that learn to detect patterns by themselves (i.e., unsupervised learning). For instance, algorithms might learn by themselves to discriminate women during the recruitment process (e.g., Dastin, 2018; Lambrecht and Tucker, 2019) without the developers or users being aware of this bias. To illustrate, an algorithm trained to select the best candidates for a job taught itself (i.e., based on the data fed to the algorithm) to discriminate against women during the recruitment process (Dastin, 2018). The algorithm extracted a rule based on the data it was fed (e.g., it detected a connection made between best candidates and males) and used the rule to make future judgments. This led Amazon to stop using its automated recruitment system. In the same vein, algorithms developed to attract new talents for STEM job opportunities targeted more men than women (Lambrecht and Tucker, 2019). As pointed out by Kleinberg et al. (2018), the training data might be “rooted in past discrimination” (Kleinberg et al., 2018, p. 116). Since the input data were biased, the output data were also biased.

Therefore, before using any established algorithms, researchers need to know what data the algorithm has been trained on to tentatively estimate the risk of bias. For example, if an algorithm has been trained to predict friendliness on videos showing mainly males from an individualistic culture, it is possible that the developed system will not offer accurate predictions for women or individuals from a collectivistic culture. In the same vein, researchers showed that algorithms trained on videos featuring only adults were biased in performing emotion recognition on a younger population (Howard et al., 2017). Researchers interested in developing their own algorithms also need to be critical about the input and output data used and created by their nonverbal social sensing system.

Biased decisions have important ethical ramifications. First, in the examples related to biased recruitment, the decision was made by a machine and not a human (see recommendations for trustworthy algorithms, High-Level Expert Group on Artificial Intelligence (AI HLEG), 2019). Second, the algorithm ended up taking into account a feature protected by law (e.g., gender and ethnicity) to produce a decision that disadvantages the said group. Given that this subject is not the main focus of this study, we referred the reader to Kleinberg et al. (2018) for a discussion on the legal and ethical aspects of discrimination associated with the use of algorithms in the recruitment process and to Raghavan et al. (2020) for potential solutions and challenges.

### Data Privacy

Another ethical issue is linked to data privacy. Social and computer scientists might not share the same ethical guidelines when studying NVB. This difference might be aggravated by open-science policies. For instance, social scientists, studying NVB based on video recordings of participants, need to ensure the anonymity of the participants and to disclose the specific use of the collected data. Meanwhile, computer scientists might not be required to do the same and to obtain the consent of participants to reuse their data. In this context, sharing data or developing corpora useful for future studies might be more difficult to achieve for social scientists than for computer scientists. Still, following the Facebook-Cambridge Analytica scandal, an ethical crisis related to data protection has also shaken computer scientists. In this context, researchers need to be attentive to ethical compliance across fields of research. In this vein, fostering collaborations between social and computer scientists might help in determining ethical guidelines that are common to both fields.

Concerning ethical algorithms, we suggested that social scientists, interested in the use of nonverbal social sensing systems, should be well-informed about policies related to artificial intelligence (AI). For instance, in Europe, a group of experts was commissioned to work on ethical guidelines for AI (Biel et al., 2013). The requirements for the so-called trustworthy AI are (1) human agency and oversight, (2) technical robustness and safety, (3) privacy data and governance, (4) transparency, (5) diversity, non-discrimination, and fairness, (6) environmental and societal wellbeing, and (7) accountability. As suggested by the High-Level Expert Group on Artificial Intelligence (AI HLEG) (2019), these seven requirements should be addressed, and reflected upon, if adherence is not feasible.

### Context-Dependency of Nonverbal Social Sensing

The quality of the output generated using nonverbal social sensing depends on the extent to which the data coded by the algorithm resemble the data on which the algorithm had been trained. To illustrate, if researchers use an algorithm that extracts head nods and this algorithm has been trained on videos featuring people sitting in front of a camera, but the video material for which the researchers want to use the algorithm shows people from the side, instead of a frontal view, involved in social interaction, it is likely that the algorithm will not perform that well.

For inferences, context-dependency is even more of an issue and the extent to which inferences are domain-specific or transversal is unclear. Will an algorithm trained to extract personality from videos of targets self-presenting during a job interview extract personality from videos of people self-presenting for a dating site with equal accuracy? Will an algorithm trained to extract trustworthiness from videos of targets giving a public speech perform equally well on videos of people answering job interview questions?

We suggested to scholars, who want to use nonverbal social sensing, to gather information about the W5 + (i.e., where, what, when, who, why, and how of the video input data the algorithm has been trained on, Vinciarelli et al., 2009b) and on potential moderators (i.e., culture, relationship, and gender, Burgoon and Dunbar, 2018). This information will enable the researcher to gauge whether the algorithm can be used for this study, as well as highlight boundary conditions or limitations of the developed algorithms for future applications.

### Off-the-Shelf vs. Tailored Approaches

Some nonverbal social sensing systems are readily available (i.e., OpenPose and OpenFace to code NVB as a unit or systems such as FaceReader to code NVB in the face as more more global judgment). These systems are easy to use for people outside the field of computer science. We thus encouraged researchers interested in coding NVB as action/motion units to try well-known off-the-shelf open-source solutions (e.g., OpenPose and OpenFace). However, researchers need to keep in mind that off-the-shelf systems might not be suited for their specific study purposes. For example, a researcher might need data on the duration of an NVB while off-the-shelf systems provide data on its frequency.

Nonverbal social sensing systems to code NVB at the inference level are also available on the market (e.g., FaceReader or Affectiva for facial expression, and HireVue and Pymetrics for hireability). These commercial off-the-shelf systems come with a caveat. They typically do not provide information about the input data (i.e., videos and ground truth) on which the algorithms have been trained, making it impossible to gauge the reliability and the accuracy of the inferences for the dataset of the researcher. To illustrate, the HireVue algorithm automatically generates a score of hireability and a rank to help companies make their hiring decisions. With this type of off-the-shelf solution, several questions arise: Does the algorithm take into account the protected features? Is human agency respected? Is the process transparent enough? How is accuracy assessed? To assess the quality of the inferences obtained by the off-the-shelf solutions, the researchers have to manually code a portion of their data and compare it with the output of the algorithm to ensure that the algorithm performs at the expected level.

Hence, when using an off-the-shelf system to code NVB at the inference level, researchers need to have access to its input and output data. This is necessary to assess its reliability and algorithm performance. Researchers are also advised to verify that the system is compliant with the existing guidelines on the use of AI (see the recommendation of OECD of the Council on Artificial Intelligence—OECD AI Principles; High-Level Expert Group on Artificial Intelligence (AI HLEG), 2019).

An alternative to the off-the-shelf solution is to become savvy in machine learning or to collaborate with computer scientists to develop an algorithm for automatic coding of NVB. These multidisciplinary collaborations can benefit both social and computer scientists by fostering the development of SSP and nonverbal social signals. Benefits have already been highlighted in the domain of neurosciences (Sedda et al., 2012). Social scientists can benefit from the technical expertise of computer scientists. Computer scientists can benefit from the expertise of social scientists in NVB studies (e.g., knowledge about taxonomies and key variables to take into account). Developing an algorithm to code for NVB is only a viable solution if the developed algorithm can be used for other research projects. This is because the generation of the input data (i.e., videos and ground truth) and the machine learning process are time and resource-intensive.

To help identify the best nonverbal social sensing approach, researchers need a clear research question. This will help them determine the type of data and method that is needed. We suggest two complementary reflections. First, the general approach to study NVB must be clarified and operationalized. In this domain, we suggest following the pragmatic guide developed by Blanch-Hartigan et al. (2018) to identify the input data and the data collection method. This step is crucial to identify whether nonverbal a social sensing system is appropriate for the research project. The questions to be answered are: Is computer vision sufficiently developed to extract the NVB? Does a model to predict global judgment already exist? and Is it necessary to create a new nonverbal social sensing system? Second, to refine choices about coding NVB decisions, we suggest that researchers clarify their coding approach (Burgoon and Dunbar, 2018). Determining NVB coding strategies directly affects nonverbal social sensing. For instance, researchers interested in kinesics at the dyadic level need at least two cameras to record each member of the dyad for data capture. Another example of a decision that needs to be taken (i.e., when, where, and by whom) concerns the granularity of the temporal dimension. To illustrate, OpenPose enables researchers to automatically code the NVB for each second of the interaction. Other issues that need to be addressed include whether an off-the-shelf solution is available to code the macro-behaviors and whether researchers are interested in objective or subjective measurements in coding NVB as a unit or an inference.

## Conclusion

Nonverbal social sensing can extract NVB from videotaped social interactions or it can make inferences based on NVB in videotaped social interactions. Both of these outputs are highly relevant for researchers, and because such algorithms allow scalability, they might attract new researchers in the domain of NVB, contributing to the advancement of the field.

However, these new technologies are still in development. Moreover, they are not free of biases and their input and output data are highly context-dependent. At this stage, ubiquitous sensing and automated extraction only complement human coding and particular caution, and scrutiny about the quality of the algorithm, needs to be taken before one can use these sensing and extraction technologies.

Researchers assessing the usefulness of nonverbal social sensing for their study should ask themselves the following questions: Can I use an algorithm that is already developed or do I have to develop my own? If I have to develop my own, do I have the necessary competencies or the necessary collaboration partners with those competencies? When using an existing algorithm: (a) Is the video input data similar to the training dataset? (b) How is the ground truth obtained? and (c) Do I know on which NVB the inferences are based? To ensure the quality and accuracy of the coding done by the algorithm on the data gathered by the researchers, said researchers might want to consider manually coding a subset of the data and then compare the performance of the algorithm with the manual coding.

The more established and robust algorithms for NVB extraction become, the more attractive they are for researchers to use and the more they might advance the field of NVB studies. This is because using established and robust algorithm for the automatic coding of NVB will improve the comparability of NVB across studies and has the potential to attract more researchers into the field.

## Author Contributions

LR, MSM, ND, and EK conceived of the presented idea. LR and MSM refined the main ideas and proof outline. All authors contributed different parts of writing to the manuscript with LR being in charge of coordinating and integrating and writing the most extensive part of the manuscript. All authors discussed the initial content and LR, MSM, and EK contributed to the final manuscript.

## Conflict of Interest

The authors declare that the research was conducted in the absence of any commercial or financial relationships that could be construed as a potential conflict of interest.

## Publisher’s Note

All claims expressed in this article are solely those of the authors and do not necessarily represent those of their affiliated organizations, or those of the publisher, the editors and the reviewers. Any product that may be evaluated in this article, or claim that may be made by its manufacturer, is not guaranteed or endorsed by the publisher.
